# Wide-gamut structural colours on oakblue butterflies by naturally tuned photonic nanoarchitectures

**DOI:** 10.1098/rsos.221487

**Published:** 2023-04-05

**Authors:** Gábor Piszter, Krisztián Kertész, Zsolt Bálint, László Péter Biró

**Affiliations:** ^1^ Institute of Technical Physics and Materials Science, Centre for Energy Research, PO Box 49, 1525 Budapest, Hungary; ^2^ Department of Zoology, Hungarian Natural History Museum, 13 Baross St., 1088 Budapest, Hungary

**Keywords:** structural colour, photonic nanostructure, *Arhopala*, butterfly, optical spectroscopy, electron microscopy

## Abstract

The iridescent structural colours of butterflies, generated by photonic nanoarchitectures, often function as species-specific sexual signals; therefore, they are reproduced precisely from generation to generation. The wing scales of oakblue hairstreak butterflies (genus *Arhopala*, Theclinae, Lycaenidae, Lepidoptera) contain multi-layer photonic nanoarchitectures, which can generate a wide range of structural colours, from violet to green. By scanning (SEM) and cross-sectional transmission electron microscopy (TEM) investigation, the colour tuning mechanism of the cover scales was explored. We revealed that the characteristic size change of structural elements in similar photonic nanoarchitectures led to different structural colours that were examined by various reflectance spectrophotometry techniques. The measured structural properties of the naturally tuned photonic nanoarchitectures were used to calculate wing reflectances, which were compared with the measurement results. We found that the simulated structural colours were systematically redshifted by 95–126 nm as compared with the measured normal-incidence reflectance results. This is attributed to the swelling of the chitinous multi-layer structures during the standard TEM sample preparation and the tilt of the cover scales, which both affect the apparent layer thicknesses in the TEM cross-sections. We proposed a simulation correction and compared the results with the layer thicknesses measured on cryogenically prepared non-embedded SEM cross-sections.

## 1. Introduction

Structural colours in insects exhibit unrivalled variety in the living world [1]. In the wide range of colour-generating photonic nanoarchitectures, it can be regularly observed in diurnal papilionoid Lepidoptera (butterflies) that at the level of individuals and in closely related species slightly modified versions of nanostructures with similar ‘blueprint' may generate unique hues of colours [2,3]. Such naturally tuned photonic nanoarchitectures were developed during the million years of evolution [4] and were optimized by playing an important role in the life of these insects [5]: structural colours are used for sexual communication [6–8], crypsis [9–11] or warning potential for predators [12]. Often these occur together, separately on the dorsal and ventral wing surfaces [13,14]. However, there are also examples where photonic nanoarchitectures have no role in the visual communication of animals, the appearance only depends on the mechanical parameters of the cuticle [15].

The photonic nanoarchitectures in butterfly wing scales are nanocomposites of two transparent media in which the refractive indices of the components are different enough and periodic in the size range of the visible light's wavelength [16]. If the refractive index ratio is suitable (chitin: approx. 1.56 [17], air: 1), certain wavelength ranges cannot propagate in the structure, they are reflected from its surface [18]. The light passing through the structure is absorbed in pigment molecules (e.g. melanin [19,20]), therefore the observer's eyes only perceive the intense reflected structural colour. Photonic nanoarchitectures can be spatially periodic in one [17,21], two [22] or three dimensions [13,23], and photonic amorphous structures also exist [24]. In many cases, special structural features can be observed that may change the optical properties, like eliminating the angular dependence of colour and transforming the specular reflectance into a more diffuse one. In the case of multi-layer-type photonic nanoarchitectures, a great variety can be found in insects, e.g. defects in the structures [15], discontinuous multi-layer structures (i.e. *Morpho*-type structures [3,25]), perforation or nanopores in the chitin matrix [2,26,27], or curvature of whole wing scales to facilitate better angular visibility of the signalling colours [28–30].

By exploring the naturally tuned photonic nanoarchitectures in closely related butterfly species, systematic knowledge may be gained that can be used in the design of the future artificial photonic structures starting from a basic form and adding small modifications based on the findings in nature to tune their optical properties. We investigated the structural colour of oakblue hairstreak butterflies (genus *Arhopala*), in which the males have iridescent dorsal wing coloration over a range from violet to green. The optical properties of the cover scales were measured using bifurcated normal-incidence probe, integrating sphere, spectrogoniometry and microspectrophotometry techniques. The properties of the photonic nanoarchitectures in the cover scales were examined by scanning (SEM) and cross-sectional transmission electron microscopy (TEM), and the measured structural data were used to calculate wing reflectances. We found that the simulated structural colours of the naturally tuned photonic nanoarchitectures were systematically redshifted by 95–126 nm compared with the normal-incidence reflectance results. This can be explained by the swelling of the chitinous multi-layer structures during the standard TEM sample preparation and the curvature or tilt of the cover scales that affects the apparent layer thicknesses in the prepared cross-sections, for which we proposed a simulation correction and compared the results with the layer thicknesses measured on cryogenically prepared non-embedded SEM cross-sections.

## 2. Results

**Figure 1.**
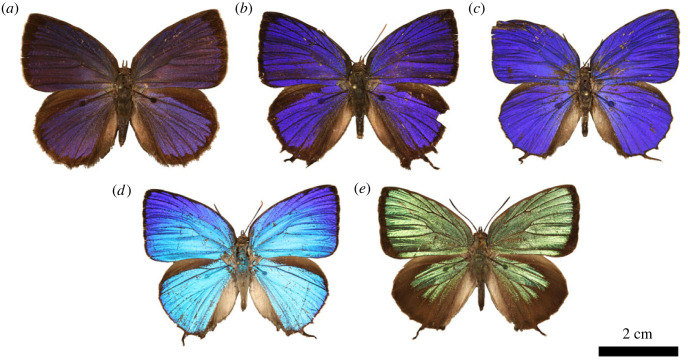
Photographs of pinned, set and dried male *Arhopala* specimens in dorsal view. (*a*) *Arhopala asopia*, (*b*) *A. nobilis*, (*c*) *A. tephlis*, (*d*) *A. araxes*, (*e*) *A. eumolphus* are shown.

**Figure 2.**
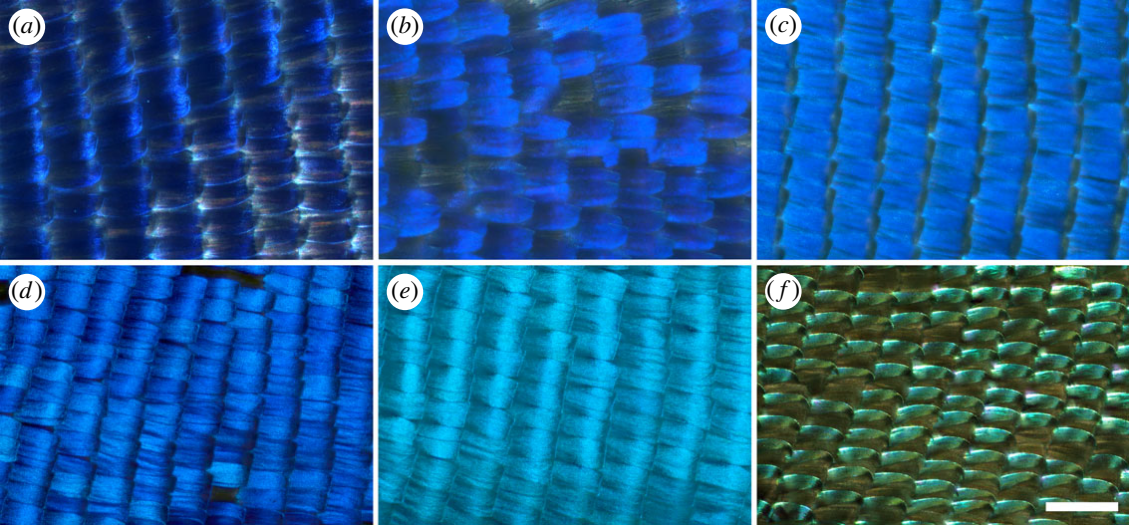
Optical microscope images of the dorsal forewing surfaces of male *Arhopala* specimens using focus stacking. (*a*) *Arhopala asopia*, (*b*) *A. nobilis*, (*c*) *A. tephlis*, (*d*) *A. araxes* forewing apical region, (*e*) *A. araxes* forewing discal region*,* (*f*) *A. eumolphus* are shown. Scale bar: 200 µm.

The males of the investigated *Arhopala* species: *A. araxes*, *A. asopia*, *A. eumolphus*, *A. nobilis*, *A. tephlis* have iridescent structural colours on their dorsal (figure 1) and pigmentary ground colours and pattern on the ventral wing surfaces (electronic supplementary material, figure S1). Optical microscope images were taken from the dorsal wing surfaces which show the arrangement of the cover scales (figure 2). In some cases, the scales have conspicuous curvature along their longitudinal and cross-sectional axes (electronic supplementary material, figure S2), which may affect their reflectance properties [31].

**Figure 3.**
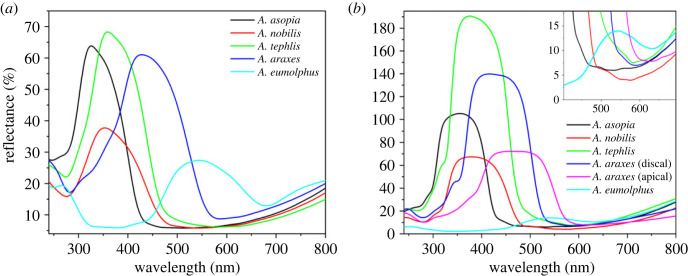
(*a*) Integrating sphere and (*b*) normal-incidence reflectance measurements of male *Arhopala* specimens. The inset shows magnified part of the graph where the relatively low amplitude reflectance of *A. eumolphus* male can be seen.

Integrating sphere and normal-incidence reflectance measurements were taken on the wings of the five investigated specimens (figure 3) using a PTFE-based diffuse tile as a reference. While the integrating sphere collects all light reflected to the upper hemisphere from the sample, the bifurcated normal-incidence probe illuminates and collects light from a spot a few millimetres in diameter. This latter method is also sensitive to the angle-dependent reflectance, therefore wings which have strong specular reflectance may reflect more light than the reference tile (resulting in greater than 100% amplitude), while wings with curved cover scales (see *A. eumolphus* scales in electronic supplementary material, figure S2) may show lower overall amplitude compared with the species with relatively flat scales. In figure 3*b*, the normal-incidence reflectance of *A. eumolphus* can be seen magnified in the inset, as the bifurcated probe could not collect the scattered light from the curved scales which resulted in the low reflectance amplitude.

**Figure 4.**
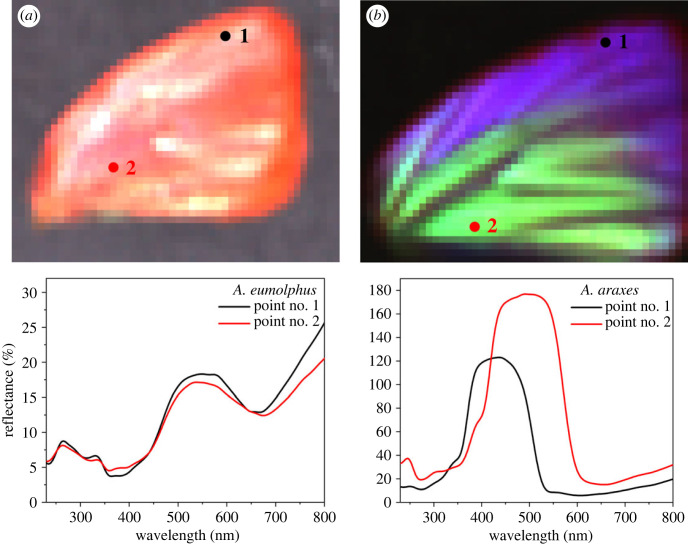
Hyperspectral imaging reflectance measurements of male (*a*) *A. eumolphus* and (*b*) *A. araxes* specimens. The lower panels show individual spectra from the selected points of the maps.

As it can be seen in the photographs (figure 1), *A. araxes* males have a gradient structural colour that shifts from the light blue (discal area) to darker blue (apical area), which were measured separately using the normal-incidence probe (figure 3*b*). To reveal further details of the colour gradient, hyperspectral imaging was used to map the wing surface by scanning the bifurcated normal-incidence probe above the sample using a motorized stage [32]. The resulting spectral array (50 × 40 pixels, total 2000 saved spectra for both wings) was analysed and depicted in the form of a false colour map to represent the colour composition of the sample. The reflectances measured on the gradient forewing of *A. araxes* compared with *A. eumolphus* with uniform structural colour can be seen in figure 4. Hereafter, the structural colour and the wing scales of *A. araxes* will be only investigated in the discal cell region, where the hue of the wing was found to be consistent.

**Figure 5.**
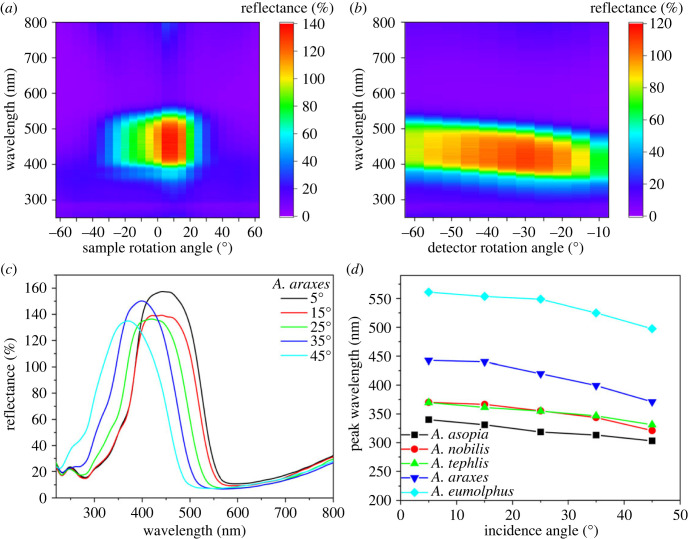
Results of the different configurations of spectrogoniometry for *A. araxes*: (*a*) back-scattered with rotating sample, (*b*) forward-scattered with rotating detector, and (*c*) specular under various incidence/detection angles. (*d*) The peak shift results of the specular reflectance measurement are shown for the investigated species.

The angle-dependence of the wings was explored using spectrogoniometry. As the reflectance of the wings of *Arhopala* specimens is highly dependent on the illumination and detection angle, three types of measurements (electronic supplementary material, figure S3) were performed [13,32,33]: back-scattered with rotating sample (electronic supplementary material, figure S4), forward-scattered with rotating detector (electronic supplementary material, figure S5) and specular under various incidence/detection angles (electronic supplementary material, figure S6). In figure 5*a–c*, the results of the three measurement configurations are depicted, respectively, for *A. araxes* as an example, while in figure 5*d,* the peak shift results of the specular reflectance measurement are summarized for the five investigated species.

**Figure 6.**
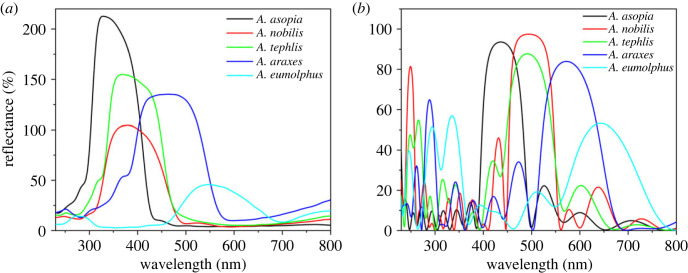
(*a*) Reflectance spectra normal to the multi-layer nanostructures of the investigated *Arhopala* species which were measured using the back-scattered spectrogoniometry set-up (electronic supplementary material, figure S3b), where the peak reflectances were determined by rotating the samples around the vertical axis. (*b*) Calculated normal-incidence spectra of the multi-layers of the male *Arhopala* specimens.

Using the back-scattered spectrogoniometry set-up, we were able to eliminate the effect of angular offset between wing and scale orientation by rotating each sample around the vertical axis to obtain maximal wing reflectances. We consider these spectra to be the normal to the multi-layers, as peak reflectance is measured at normal incidence (figure 6*a*).

**Figure 7.**
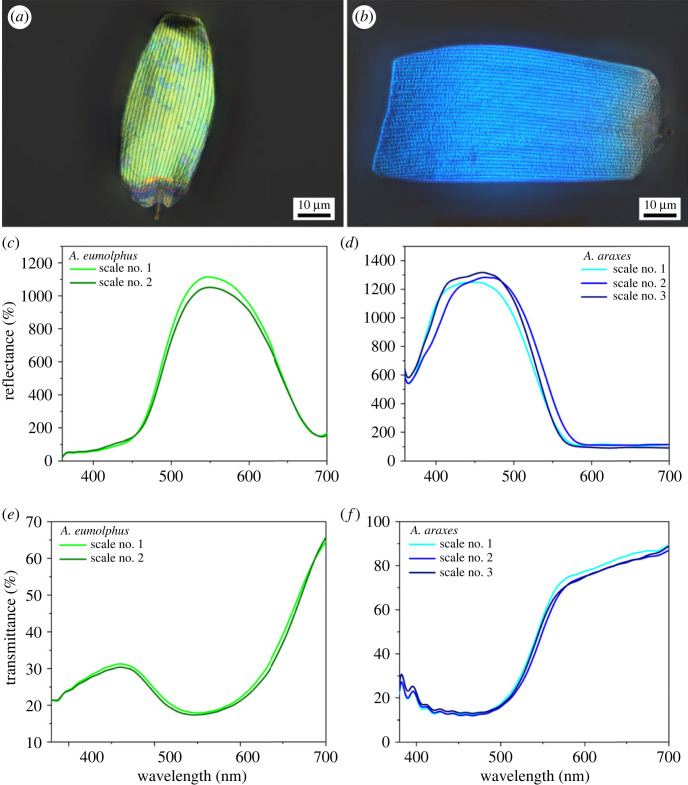
Single scale micrographs (top), reflectance (middle) and transmittance spectra (bottom) of *A. eumolphus* (*a*,*c*,*e*) and *A. araxes* (*b*,*d*,*f*) cover scales are shown.

Microspectrophotometry of single separated wing scales was conducted on selected specimens of *Arhopala* butterflies. As the set-up was based on a standard optical microscope, where the UV transmittance was limited below 380 nm, only two species were investigated, both of which had distinct reflectance peaks in the visible spectral range (see *A. araxes* and *A. eumolphus* in figures 3 and 6*a*). Using this method, the differences which may arise from the cover scale orientation or from the coloration of ground scales/wing membrane were eliminated. Reflectance and transmittance measurements were conducted on the cover scales of both species with respect to the reflectance and the transmittance of the glass slide substrate, respectively. In the case of *A. eumolphus* two, in *A. araxes* three single scales were removed from the discal cell region, and the spectral properties were measured near the middle of the scales where the colour was the most homogeneous. For a given species, both reflectance (figure 7*c,d*) and transmittance (figure 7*e,f*) spectra of the cover scales were found to be almost identical.

**Figure 8.**
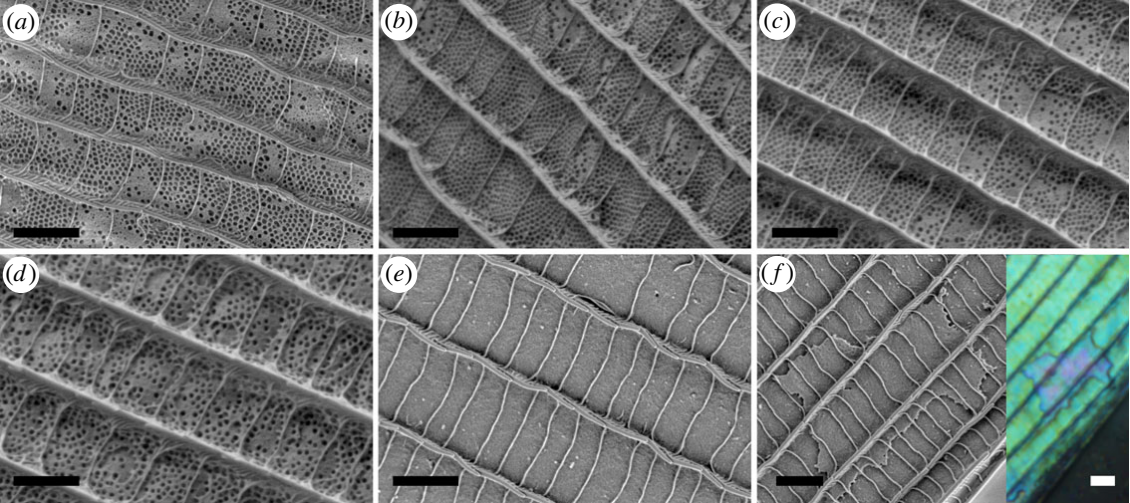
SEM images of the cover scales of the investigated species. (*a*) *Arhopala asopia*, (*b*) *A. nobilis*, (*c*) *A. tephlis*, (*d*) *A. araxes*, (*e*) *A. eumolphus* are shown. (*f*) SEM and optical microscope images of the same additional chitinous layer occurring on *A. eumolphus* cover scale. Note the colour change due to the additional layer in the optical image. Scale bars: 2 µm.

**Figure 9.**
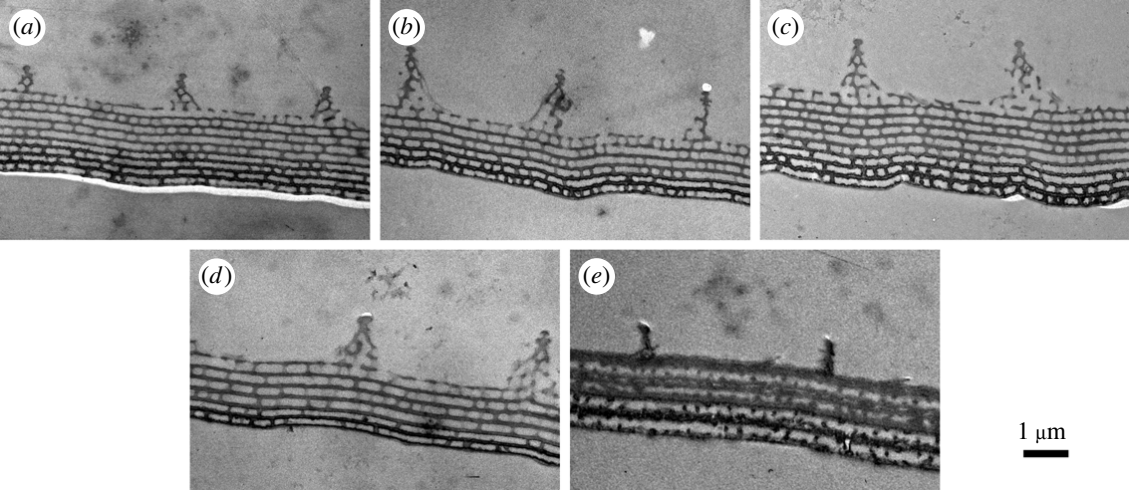
Cross-sectional TEM images of the cover scales. (*a*) *Arhopala asopia*, (*b*) *A. nobilis*, (*c*) *A. tephlis*, (*d*) *A. araxes*, (*e*) *A. eumolphus* are shown.

SEM (figure 8) and cross-sectional TEM (figure 9) images of the cover scales were taken for the five species. The TEM revealed the multi-layer-type structure of the photonic nanoarchitecture, characteristic for the genus, while in the SEM images nanoporous upper chitinous layer(s) can be observed. Based on TEM images, the layer thicknesses of both chitinous and air-rich layers were measured for all specimens in 10 images, and the results were averaged (electronic supplementary material, table S1). Using a standard atomic force microscope (AFM) in contact mode and scanning the tip at a constant height over the scale, we were able to remove the ridges and cross-ribs from the cover scale surface, thus revealing the whole underlying nanoporous structure [34]. When the distance between the tip and the scale was further decreased, the chitinous pillars supporting the multi-layer structure were revealed (electronic supplementary material, figure S7). Based on these SEM image series and the measured geometries, the refractive indices of the chitinous and air-rich layers were estimated. As the chitinous layers contain air-filled nanopores and the air-rich layers are supported by chitinous pillars, the refractive index contrast was smaller than the ‘ideal' 1.56 : 1 [8]; in our case, we estimated 1.4 : 1.1 from the weighted average of the structural elements for the investigated species.

Based on the measured layer thickness and refractive index data, optical models of the five species were generated and a transfer matrix method-based analytical calculation [32,35] was used to simulate the reflectance spectra of the investigated photonic nanoarchitectures. Figure 6*b* depicts the calculated normal-incidence reflectances of the five multi-layers based on the SEM and cross-sectional TEM data.

**Figure 10.**
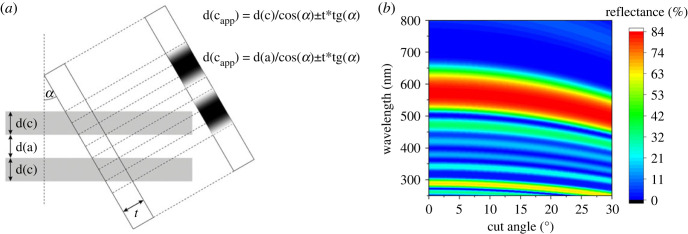
(*a*) Schematic of the oblique angle cut on a structure of parallel chitin/air layers. (*b*) Calculated normal-incidence reflectance spectra of *A. araxes* respective to the scale surface, where the layer thickness is determined on TEM images at different cut angles.

Despite the careful optical measurements and thorough electron microscopy analysis, we found a significant difference (table 1) between the measured structural colours (figure 6*a*) and the simulated reflectances (figure 6*b*). This may be attributed to the swelling of the chitinous multi-layer structures during the standard TEM sample preparation [36,37], the not perfectly normal orientation of the cross-sections through the scales (oblique angle cut of the cover scales) during the ultramicrotome slicing and the natural tilt of the scales attached to the wing membrane (the plane of the wing membrane is taken as a reference plane, when sectioning to TEM samples). To examine the latter effect of the oblique angle cuts and calculate the possible inclination of the experimentally used 70 nm thick wing scale sections, we modified the multi-layer simulation in the following way. By taking a parallel multi-layer built from chitin and air and supposing a sectional cut with a thickness *d* at an angle *α* respective to the normal of the layers, we observed an increase of the apparent layer thickness for chitin, *d*(*c*_app_) and air, *d*(*a*_app_) (figure 10*a*). In the case of the cut, transitional ranges appear at the upper and lower part of the solid chitinous layer. The boundaries of the layers are not visually well defined in the TEM sections (figure 10) due to the off-axis imaging and the contrast settings of the TEM. Therefore, the user measuring the layer thickness on TEM micrographs has to decide about the transitional range: assigning it to the chitin or air part has significant influence on the outcome of the optical simulation. To quantify the effect of the section angle on the calculated reflectance spectra, figure 10*b* as an example for *A. araxes*, where half of the transitional range is assigned to chitin and half to air as an intermediate example.

**Table 1.** Peak wavelength values of the measured and simulated wing reflectances and their differences for the investigated *Arhopala* species.

species	measured reflectance [nm]	TEM-based simulation [nm]	Δ_T-M_ [nm]	SEM-based simulation [nm]	Δ_S-M_ [nm]
*A. asopia*	328.7	436.0	107.3	313.0	−15.7
*A. tephlis*	367.8	494.0	126.2	325.0	−42.8
*A. nobilis*	379.7	491.0	111.3	299.0	−80.7
*A. araxes*	463.2	572.0	108.8	301.0	−162.2
*A. eumolphus*	548.3	643.0	94.7	540.0	−8.3

## 3. Discussion

The genus *Arhopala* (Lycaenidae: Theclinae: Arhopalini) is distributed in the tropical and subtropical regions of Asia and Australia. There are more than 200 species recognized, their caterpillars are mainly hosted by dicotyledon plants, chiefly Fagales [38,39]. The imagines live in the forest canopy. Contrasting to their relatives classified in the subfamily Theclinae, the tribe Arhopalini does not possess any male secondary characters [38,40]. Therefore, *Arhopala* species have a wide range of physical colours, from violet to green (figures 1 and 2). These are generated by nanoporous multi-layer photonic nanostructures which are located in the lumen of the cover scales (figures 8 and 9). The structural colours are important in the life of these insects, as they are used as sexual signalling colours [41,42], therefore their effective perceptibility is crucial. It seems likely that the changes in the wing scale nanoarchitectures have resulted from evolutionary pressure driven by perceptibility. For example, changes in scattering [43], disorder [2] or scale curvature [28,29,30] (figure 2; electronic supplementary material, figure S2) all increase the angular range over which colour is reflected.

Using normal-incidence reflectance spectroscopy (figure 3*b*), a reflectance peak was measured for most of the investigated species, characteristic of multi-layer nanoarchitectures [44]. In the case of *A. eumolphus*, a fraction of the reflectance amplitude was measured compared with others (see the inset of figure 3*b*), which is due to the fact that the wing scales with significant curvature (see electronic supplementary material, figure S2) reflected most of the normal-incidence light in directions other than normal to the wing membrane, which were not detected by the probe [45]. The integrating sphere offers a solution for this problem, as the spectra measured in this way are collected from a relatively large spot (5 mm in diameter), which may contain hundreds of cover scales, thus integrating their angle-dependent reflectance (figure 3*a*). The structural colour of *A. asopia* falls in the near-UV wavelength range, therefore human eyes—and digital cameras with similar spectral sensitivity (greater than 400 nm)—can only see a small fraction of the reflected light due to their limited spectral sensitivity (figure 1*a*). Butterflies are able to detect these near-UV wavelengths as their eyes are often supplemented with additional visual pigments outside the human visible range [46]. The other two blue species, *A. nobilis* and *A. tephlis*, have partial UV colour, and on the forewings of *A. araxes*, a gradient can be seen in the structural colour (figure 3*b*).

Using hyperspectral imaging, the gradient can be measured and mapped in detail (figure 4). In the case of a monochromatic species (*A. eumolphus*), only minor local differences were found, despite the fact that normal-incidence measurement may be sensitive on the texture of the wing [47], while for *A. araxes*, the maximum shift of the reflectance spectrum reached 100 nm through the forewing. This variability of the colour has to be taken into account; therefore, all other spectral and physical investigations were carried out in the discal cell area of the wing where the most consistent colour was measured (point 2 in figure 4*b*).

Individual wing scales were separated and investigated by microspectrophotometry (figure 7). We found that the reflectance of the single scales was highly reproducible and matched well with the entire wing's reflectance measured macroscopically (figures 3 and 6*a*), as the wavelength of the peak was approximately 550 nm for *A. eumolphus*, while it was approximately 450 nm for *A. araxes*. These show that it is appropriate to compare the result of the microscopic optical simulation with the macroscopic normal-incidence measurements. Since the spectrophotometer was connected to a standard optical microscope, the optical elements of which were made of inherently UV-absorbing glass, only species reflecting entirely in the visible wavelength range could be measured properly.

The cover scales of the *A. eumolphus* specimen showed deviations from the typical green colour in some places, where a different hue was visible. The reason for this was found to be an additional chitinous layer of the photonic nanoarchitecture, which resulted in a locally shifted structural colour. We were able to observe the layers in the nanostructure using both an SEM and an optical microscope (figure 8*f*). These did not alter the wavelength position of the main reflectance peak, but the amplitude ratio change of the main peak and the satellite peaks resulted in different colour locally (figure 8*f*).

The angle-dependent reflectance of the wings was investigated using three configurations (electronic supplementary material, figure S3) of a spectrogoniometer. Forward-scattering is useful for showing that although structural colours are visible over a wider angular range compared with a perfect multi-layer, they still cannot be considered diffuse, Lambertian reflectors. The reflected light was typically detected in the range of 20–40° while rotating the wing, not only in specular reflection (electronic supplementary material, figure S5). This may be caused by the nanopores in the multi-layer [2,26,27], the scattering effect of the ridges [43], and also by the curvature of the cover scales [28,29]. With the specular reflection measurement set-up (figure 5*c*; electronic supplementary material, figure S6), the shift of the structural colour as the function of the illumination/detection angle can be shown. This was compared for the five species by finding the peak wavelength for all rotation angles and plotting them together in figure 5*d*. An almost identical linear behaviour was found for the five species, showing that their multi-layer photonic nanoarchitectures are highly similar; only the tuning of the structural colour, i.e. the typical vertical dimensions of the multi-layer photonic nanoarchitectures were different. Finally, the back-scattered set-up can be used effectively to estimate predominant plane of the scales with respect to the wing membrane, as the maximum reflected intensity is shifted from the normal to the wing membrane with that angle (electronic supplementary material, figure S4). Furthermore, this method allows us to measure the reflectance spectra normal to the multi-layers, where the angle of the cover scales attached to the wing membrane (typically 15–25°) is compensated.

Optical simulations were conducted to compare the known normal incidence reflectances, rather than the reflectance of the less well-defined/unknown angle of the wing scales. Therefore, reflectance spectra normal to the multi-layer structures were calculated (figure 6*b*) and also measured using the back-scattered spectrogoniometry set-up (electronic supplementary material, figure S3b), where the peak reflectances were determined by rotating the samples around the vertical axis (figure 6*a*). We found that the simulated structural colours of the naturally tuned photonic nanoarchitectures were systematically redshifted by 95–126 nm compared with the measured normal-incidence reflectance results (table 1). Apart from the approximately 100 nm redshift on average, the relative positions of the spectra were matched within ±12 nm standard deviation in the measured (figure 6*a*) and simulated (figure 6*b*) cases, which indicates a systematic structural deviation of the TEM sections.

This can be explained by the uniform swelling of the cross-sections during the TEM sample preparation and by the curvature and angle of the cover scales attached to the wing membrane that affect the apparent layer thicknesses when the sections were prepared by perpendicular cuts to the wing plane, respectively (figure 10). The swelling of the photonic nanoarchitectures occurs during the steps of embedding the wing pieces into the resin during the TEM sample preparation and results in uniformly increased layer thicknesses measured on the sections. In the literature [36,37], swelling of the cuticle has been observed when sections were prepared from insects for electron microscopy.

Due to the variation of the embedding angle of the wing pieces and angle of attack differences of the wing scales combined may result in an additional deviation of the characteristic sizes of the photonic nanoarchitectures measured in the TEM images [48]. By modelling the effect of the oblique angle sections, we were able to compensate for the additional spectral shift of the calculated reflectance spectra beyond swelling (figure 10). The magnitude of the correction was comparable to the experimentally determined when 15–25° cover scale angles (figure 5*a*; electronic supplementary material, figure S4) were used in the simulations as input: a maximum of 50 nm spectral shift was covered by this calculation in the case of *A. araxes*, where the actual shift was more than 100 nm resulted from the swelling and the oblique angle cut together. Similar differences were observed between the correction and the measured shift for the other investigated species, suggesting a approximately 10% uniform swelling of the photonic nanoarchitectures during TEM sample preparation. Although the structural details of the nanoarchitectures remain, the exact measured dimensions have to be handled carefully. The differences arising from the above-mentioned uncertainties and from the natural variability of the biological samples can be handled by control experiments and statistical analysis of a large number of samples together [32,47], but this requires considerable amount of resources.

To investigate the swelling of the photonic nanoarchitectures during the resin embedding, sections were cryogenically prepared from the cover scales of the investigated species. The thickness of the chitinous and air layers were measured on SEM images taken from the surface of the obtained sections (electronic supplementary material, figure S8a), from which model structures were generated for the optical simulation. The calculated spectral results (electronic supplementary material, figure S8b) clearly show that photonic nanoarchitectures in the sections prepared without embedding are not swollen. In fact, the significant blueshift indicates that the multi-layer nanostructures were damaged to a greater extent due to the compression during the cryogenic sectioning as compared with the TEM sample preparation. When the non-embedded scales were sectioned, all the load has to be supported by the nanopillars (trabeculae) separating the chitinous layers. This resulted in the blueshift and the higher variation of the calculated reflectances (table 1): while *A. asopia* and *A. eumolphus* had almost identical calculated reflectances with the measured ones, the other three species showed differences in the peak wavelength ranging from 40 to 160 nm. The results above imply that it is worth using the high-precision ultramicrotome slicing and resin embedding for the TEM for the structural characterization of photonic nanoarchitectures, even when careful examination and numerical corrections are required after the sample preparation.

## 4. Conclusion

The structural colour of the males of five hairstreak butterfly species belonging to the genus *Arhopala* was investigated using various reflectance spectroscopy and microscopy techniques. We found that the wide-gamut structural colours of the dorsal wing surfaces, from violet to green, were generated by multi-layer photonic nanoarchitectures located in the lumen of the cover scales. Using cross-sectional TEM and SEM imaging, the structural parameters of the cover scales were measured, and the obtained data were used to simulate the reflectance of the naturally tuned multi-layer structures. The results of the simulation were compared directly with the reflectance spectra measured using a back-scattered set-up of a spectrogoniometer which compensated the angle of the cover scales attached to the wing membrane. The calculated spectra based on the cross-sectional TEM data were significantly redshifted compared with the measured results, while the calculated spectra from the SEM data blueshifted. This can be explained by the swelling of the photonic nanoarchitectures, which occurred only during the sample preparation required for TEM, and it can also be caused by the oblique angle cut of the wing scales during ultramictrotome sectioning. To eliminate the resulting apparent size change of the photonic nanoarchitectures, we proposed a simulation correction and compared the results with the layer thicknesses measured on non-embedded SEM cross-sections.

## 5. Material and methods

### 5.1. Butterflies

The butterfly samples were obtained from the curated collection of the Institute of Technical Physics and Materials Science, Centre for Energy Research. Male specimens of *A. araxes, A. asopia, A. eumolphus, A. nobilis*, and *A. tephlis* (Lycaenidae: Theclinae: Arhopalini) were investigated (electronic supplementary material, table S2). None of the species used in this study were subjected to any restrictions.

### 5.2. Photography and optical microscopy

Photographs were taken using a Canon EOS 5D Mark III (Tokyo, Japan) digital camera with halogen light illumination. Optical microscopy was carried out using ×20 and ×100 objectives of a Nikon Eclipse LV150N (Shinagawa, Tokyo, Japan) device with extended depth of focus (EDF) mode which resulted in high depth of field images of the otherwise textured butterfly wing surfaces.

### 5.3. Scanning electron microscopy, transmission electron microscopy and atomic force microscopy

The butterfly wing samples were prepared for electron microscopy using standard techniques [2]. Cryogenically prepared wing scales were cut in liquid nitrogen using a surgical prep blade. The samples were examined via scanning electron microscopy (SEM) and cross-sectional TEM imaging using Thermo Fisher Scientific Scios 2 DualBeam (Waltham, MA, USA) and Philips CM20 (Eindhoven, The Netherlands) systems, respectively. For TEM, a standard sample preparation procedure was performed: after fixing the few millimetres long wing pieces for 45 min in 2.5% glutaraldehyde and 2% formaldehyde in 0.1 M Na-cacodylate buffer (pH 7.2), the specimens were rinsed in 0.1 M Na-cacodylate and distilled water and were dehydrated in graded ethanol (50–100% in 10% steps). Next, they were infiltrated with propylene oxide and embedded in Spurr's resin (SPI Supplies, West Chester, PA, USA). Ultrathin sections were made with a diamond knife (Histo, Diatom, Switzerland), double-stained with uranyl acetate and lead citrate and observed with a transmission electron microscope at an accelerating voltage of 200 keV.

AFM lithography was performed with a Veeco Multimode Nanoscope V SPM (Billerica, MA, USA) under ambient conditions. A silicon scanning tip (Nanosensors, Neuchatel, Switzerland: force constant 50 N m^−1^, radius less than 25 nm) was moved in contact mode at a constant height with a tip velocity of 20 µm s^−1^ over the cover scales. We were able to remove the ridges, cross ribs and upper layers from the scales by gradually decreasing the distance between the tip and scale surface.

### 5.4. Reflectance spectrophotometry

Spectral measurements were carried out using an Avantes (Apeldoorn, The Netherlands) fibre-optic system consisting of an AvaSpec-HSC1024×58TEC-EVO spectrometer and an AvaLight-DH-S-BAL light source. Reflectance of the butterfly wings was measured using a normal-incidence bifurcated probe (FCR-7UV200-ME-SR) and integrating sphere (AvaSphere-30-REFL) with a WS-2 diffuse tile as a reference.

For detailed mapping of the reflectance spectra over the entire wing surface, a custom-made set-up was used based on an Optics Focus (Beijing, China) Motorized XY-Axis Stage and a normal-incidence bifurcated probe attached to the spectrometer [32]. A custom LabView (Austin, TX, USA) application was used to synchronize the movement of the stage and storage of the data from the spectrometer.

Single wing scale measurements in reflected and transmitted light were conducted by connecting the Avantes spectrometer to a Zeiss Axio Imager A1 optical microscope (Jena, Germany) with × 100 Epiplan objective through a custom-made adapter tube and optical fibres [32]. The spot size was less than 10 µm using this objective. The reflectance and transmittance were measured close to the middle of the wing scales or where the colour was the most homogeneous (figure 7*a*). Reflectance and transmittance measurements were conducted with respect to the reflectance and transmittance of the glass slide substrate, respectively.

To investigate the angular dependence of the wing reflectance, we used a set-up consisting of a custom-made goniometer and fibre optics with collimating lenses [13,32]. The different configurations can be seen in electronic supplementary material, figure S3.

Data analysis and plotting were performed in OriginPro 2021 (OriginLab Corporation, Northampton, MA, USA) software.

### 5.5. Optical simulation

The photonic nanoarchitectures of the wing scales were modelled as chitin–air multi-layer structures using transfer matrix method [35,49,50]. The thickness of chitinous and air-rich layers of the cover scales was measured on the cross-sectional TEM images in multiple points. These values were averaged and used as input in the simulations (electronic supplementary material, table S1). The refractive indices of the layers were estimated based on the SEM images after the AFM lithography. Reflectance spectra were calculated for normal-incidence white light (figure 6*b*) and were compared with the measured wing reflectances (figure 6*a*).

## Data Availability

All relevant data analysed during this study are included in this published article and its electronic supplementary material files [51]. Raw datasets used during the current study are available from the corresponding author on reasonable request.
